# *Helicobacter pylori* and Gastric Cancer

**DOI:** 10.3389/fmed.2016.00036

**Published:** 2016-08-22

**Authors:** Amin Talebi Bezmin Abadi

**Affiliations:** ^1^Department of Bacteriology, Faculty of Medical Sciences, Tarbiat Modares University, Tehran, Iran

**Keywords:** *Helicobacter pylori*, gastric cancer, research directions

## *Helicobacter pylori*: State of the Art

Existence of any infectious agents in stomach was a big query among the microbiologists and gastroenterologists (1–3). *Helicobacter pylori* (*H. pylori*) is a leading cause of severe digestive disease, including gastric cancer (4, 5). Although over the last years, incidence of gastric carcinoma is decreasing worldwide, it remains the fourth most common cancer and the second most common cause of malignancy-related death worldwide (6, 7). During the years after *H. pylori* discovery, especially following many meta-analyses and certain number of experimental evidences, it became a crucial question whether there is a link between gastric cancer and *H. pylori* infection (8). A contributory role for *H. pylori* in gastric cancer was first announced by the International Agency for Research on Cancer (IARC), when they labeled *H. pylori* a class I carcinogen (9). Moreover, since the prevalence of *H. pylori* and pattern of superficial gastritis can change quickly within a specific population, the incidence of gastric cancer can also shift rapidly (10). It has been generally accepted that the risk of cancer is highest among patients in whom the primary colonization causes acute and then chronic inflammation (11). To our knowledge, certain *H. pylori* strains seem to differently increase the risk of cancer, depending on the existence of certain bacterial genotypes (for example: *cagA*) (12, 13). Bacterial-secreted CagA, inducing high levels of chronic inflammation, is the main factor increasing mutagenesis rate, oxidative-stress, and increased mismatch repair pathways, resulting in gastric carcinogenesis (14, 15). Hence, both prevalence and incidence rates are different in various geographical areas (16, 17). Taking together, in this paper, we aim to point out all important topics concerning management of *H. pylori* strains associated with gastric cancer.

### Prevention of Gastric Cancer

To now, prevention is an optimal approach to deal with gastric cancer. Thus, all choices ending in elimination of the *H. pylori* would be on the desk. Unfortunately, we do not have any preventive or therapeutic vaccine against the infection, but ongoing research is trying to find the best solution (18). Using metagenomic experiments can help scientists to determine properties of each microbiota member, which contributes in pathogenesis of gastric cancer. The molecular mechanism of the interaction between gastric epithelial cells and *H. pylori* may also suggest a novel strategy for effective prevention of the development of gastric cancer (19, 20). Considering genetical background bound to high risk of gastric cancer, it is a major recommendation to eradicate *H. pylori* in persons with a family history of gastric cancer (21, 22). Further studies are necessary to elucidate actual role of *H. pylori* as causative agent to the development of gastric cancer.

### *H. pylori* and Antibiotic Resistance

Overall, *H. pylori* resistance to antibiotics, including clarithromycin and metronidazole, has increased during the last years; new therapeutic regimens are required in both national and global levels (23). Although a large number of therapeutic regimens are available, none had proven to be superior. Thus, effective country-based antibiotic therapy programs should be continued, especially in high prevalence regions, such as India and Iran (24–27). According to the latest Maastricht Guidelines, in regions of low clarithromycin resistance (<15%), clarithromycin-containing treatments are recommended for first-line therapy (28). So far, in regions with high resistance to clarithromycin (>15%), the quadruple treatment, including bismuth, has been proposed as first-line treatment. Sequential therapy [non-bismuth (three antibiotics plus proton pump inhibitors) quadruple therapy] was recommended in the case of unavailability of the above therapy. The third-line therapy of *H. pylori* is another challenging topic in treatment of this infection. Now, most of international guidelines suggest that patients requiring third-line therapy should be advised to the antibiotic susceptibility test before prescribing. However, an empirical therapy (such as levofloxacin-based or furazolidone-based therapies) can be applied if antimicrobial sensitivity data are not ready.

### Existence of the Virulent Bacterium

*H. pylori* infection is thought to be acquired in early childhood mostly with the fecal–oral mode of transmission (29, 30). In order to answer how only a few among those with *H. pylori* infection develop gastric cancer, it has been proposed that there are highly specific strains of *H. pylori*, called “virulent bacterium,” carrying certain genotypes of *cagA* (1). Of interest, current knowledge elucidating the etiologic role of the *H. pylori* CagA in gastric cancer is lacking (31–33).

## Conclusion

Gastric cancer induced by *H. pylori* is one of the malignancies associated with inflammation (34). Extensive epidemiologic studies showed that *H. pylori* eradication reduces bacterial effects relevant to the gastric cancer (35). The passed direction of *H. pylori* and gastric cancer research indicating on a shared line which should be well-established following next investigations. To date, existing findings indicate that gastric cancer is the biologic translation of carrying an infectious disease, which is interestingly preventive with anti-*H. pylori* regimen (36, 37). Therefore, as an inevitable consequence, identification of *H. pylori* colonized in people with high risk of gastric cancer is the main direction of the future research.

## Author Contributions

ATB suggested the idea of writing the manuscript, finalized the same, and also approved the final version before submission.

## Conflict of Interest Statement

The author declares that the research was conducted in the absence of any commercial or financial relationships that could be construed as a potential conflict of interest.
